# Targeting microRNAs to Regulate the Integrity of the Blood–Brain Barrier

**DOI:** 10.3389/fbioe.2021.673415

**Published:** 2021-06-11

**Authors:** Juntao Wang, Fang Xu, Xiaoming Zhu, Xianghua Li, Yankun Li, Jia Li

**Affiliations:** ^1^School of Nuclear Technology and Chemistry and Biology, Hubei University of Science and Technology, Xianning, China; ^2^Hubei Key Laboratory of Radiation Chemistry and Functional Materials, Hubei University of Science and Technology, Xianning, China; ^3^School of Pharmacy, Hubei University of Science and Technology, Xianning, China; ^4^Hubei Key Laboratory of Cardiovascular, Cerebrovascular, and Metabolic Disorders, Hubei University of Science and Technology, Xianning, China; ^5^Centre for Motor Neuron Disease, Department of Biomedical Sciences, Faculty of Medicine and Health Sciences, Macquarie University, Sydney, NSW, Australia

**Keywords:** blood–brain barrier, regulate, targeting, microRNA, nanobiotechnology

## Abstract

The blood–brain barrier (BBB) is a highly specialized neurovascular unit that protects the brain from potentially harmful substances. In addition, the BBB also engages in the exchange of essential nutrients between the vasculature and brain parenchyma, which is critical for brain homeostasis. Brain diseases, including neurological disorders and cerebrovascular diseases, are often associated with disrupted BBB integrity, evidenced by increased permeability. Therefore, defining the mechanisms underlying the regulation of BBB integrity is crucial for the development of novel therapeutics targeting brain diseases. MicroRNAs (miRNA), a type of small non-coding RNAs, are emerging as an important regulator of BBB integrity. Here we review recent developments related to the role of miRNAs in regulating BBB integrity.

## Introduction

The blood–brain barrier (BBB) is a multicellular neurovascular complex, mainly consisting of brain endothelial cells and supporting cells, such as pericytes and astrocytes (Profaci et al., 2020). In addition to its key role in preventing neurotoxic agents from entering the brain, the BBB also regulates the exchange of essential nutrition between the brain and the blood (Banks, 2016). The function of the BBB depends on its intact structure, or BBB integrity (Banks, 2016). Disrupted BBB integrity has been shown to contribute to the onset and progression of diseases in the brain, including neurodegenerative diseases and cerebrovascular diseases (Sweeney et al., 2018).

A large body of studies has focused on understanding how BBB integrity is regulated (Sweeney et al., 2016; Nation et al., 2019; Li et al., 2020). These efforts have led to the finding of several key molecules and singling pathways, in both brain endothelial cells and supporting cells, which are critical for the maintenance of BBB integrity, including microRNAs (miRNAs), endothelial junction molecules (e.g., VE-cadherin, claudin-5), fatty acid transporter (e.g., mfsd2a), platelet-derived growth factor (PDGF) signaling, and Wnt/β-catenin signaling (Ben-Zvi et al., 2014; Chakraborty et al., 2020; Li et al., 2020).

MicroRNAs are a class of endogenous small non-coding RNAs (20–25 nucleotides) that regulate genes at the post-transcriptional stage through either cleavage of mRNA or inhibition of translation (Chakraborty et al., 2020). In humans, approximately 2,500 mature miRNAs have been identified to regulate more than 30% of all proteins expressed in humans, suggesting the profound role of miRNAs in human physiology and pathology (Kozomara et al., 2019). Recently, the role of miRNAs in the modulation of BBB integrity has drawn great attention (Toyama et al., 2018; Chakraborty et al., 2020). In this review, we attempt to outline the involvement of miRNAs in the regulation of BBB integrity. We first briefly summarize the biogenesis and function of miRNAs, followed by a discussion of how miRNAs regulate BBB integrity by targeting different units in the BBB complex with an emphasis on the brain endothelial cells. We end by highlighting the challenges of developing efficient miRNA-based therapeutics targeting the disrupted BBB.

## Biogenesis and Function of miRNAs

MicroRNA genes, which can be either intergenic or intronic, are first transcribed by RNA polymerase II to pri-miRNAs, followed by being processed into pre-miRNAs in the nucleus (Treiber et al., 2019). Pre-miRNAs are then exported to the cytoplasm to be further processed into imperfect double-stranded RNA duplex including guide strand (miRNA) and passenger strand (miRNA^∗^). After being loaded to the RNA-induced silencing complex (RISC), the passenger strand is quickly excluded from RISC and degraded rapidly, leading to a strong preference toward the guide stand, the form of a mature miRNA (Gebert and MacRae, 2019).

MicroRNA is a posttranscriptional regulator of gene expression that contributes to diverse cellular processes, such as development, proliferation, differentiation, and apoptosis. MiRNAs recognize and bind to their target mRNAs via the seed region, a sequence of six contiguous nucleotides found from position 2–7 at the 5′-end of the molecule through direct Watson-Crick base-pairing (Gebert and MacRae, 2019). Once loaded into the RISC, the mature miRNA acts to guide the RISC to bind to partially complementary sequences within the 3′ untranslated region (UTR) of target mRNAs, resulting in the destabilization of mRNA or/and inhibition of translation (Treiber et al., 2019). For example, the first miRNA, *lin-4*, negatively regulates its target, lin-14, by repressing translation. In comparison, miR-27a regulates its target VE-cadherin at both mRNA and protein level (Young et al., 2013). It has been proposed that ancillary nucleotides at the 3′-end of the miRNA also play an important role in target recognition (Li et al., 2016).

## MiRNAs in Regulating BBB Integrity

**FIGURE 1:**
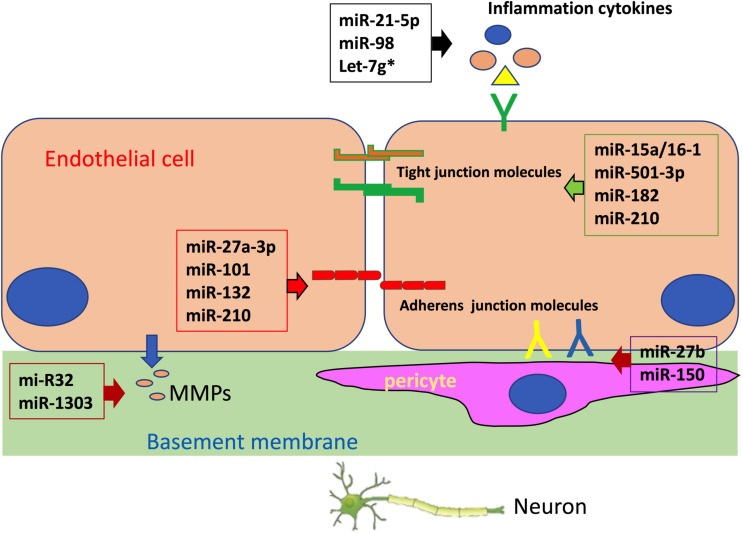
MicroRNAs (miRNAs) regulate blood–brain barrier (BBB) integrity by targeting endothelial tight junction, basement membrane, pericyte coverage, and inflammation.

The integrity of the BBB is mainly determined by brain endothelial cells, which are the fundamental unit of the BBB (Greene et al., 2020). Changes in tight junctions between brain endothelial cells and transcytosis in these cells have a significant effect on BBB integrity (Ayloo and Gu, 2019). In addition, crosstalk between endothelial cells and supporting cells forming the neurovascular unit (NVU), such as pericytes, astrocytes, immune cells, and other cells in the brain also contributes to the maintenance of BBB integrity (Profaci et al., 2020). Despite the fact that miRNAs are involved in brain diseases through various pathways (Juzwik et al., 2019; Ludwig et al., 2019; Sonoda et al., 2019; Starhof et al., 2019; Qian et al., 2020), the majority of miRNAs found to regulate BBB integrity exert their impact by targeting brain endothelial cells (Chakraborty et al., 2020). MiRNAs can either directly target endothelial junction molecules or modulate inflammation, endothelial cell survival, apoptosis, actin cytoskeleton, and other pathways to indirectly influence tight junctions in the BBB, leading to change in BBB integrity. Additionally, miRNAs may also have an impact on the crosstalk between brain endothelial cells and supporting cells, which is critical for the maintenance of BBB integrity (Figure 1).

### MiRNAs and Tight Junctions

One of the key features of the BBB is the existence of extremely tight junctions between brain endothelial cells (Vanlandewijck et al., 2018), which are controlled by a range of junction molecules, including tight junction molecules [e.g., *zona occludens* (ZO), occludin, and claudin-5] and adherens junction molecules (e.g., VE-cadherin) (Cristante et al., 2013). MiRNAs have been shown to directly target these junction molecules, leading to a change in BBB integrity (Ma et al., 2017, 2020; Zuo et al., 2019; Table 1).

**TABLE 1:** The effect of microRNAs (miRNAs) on blood–brain barrier (BBB) integrity.

Modulation of the BBB	miRNA	Target	Function	References
Tight junction	miR-15a/16-1	Claudin-5	BBB destructive	Ma et al., 2020
	miR-501-3p	ZO1	BBB destructive	Toyama et al., 2018
	miR-182	FoxO1	BBB destructive	Taddei et al., 2008
	miRNA-107	Endophilin-1	BBB destructive	Zhang et al., 2020
	miR-143	PUMA	BBB destructive	Bai et al., 2016
	miR-27a-3p	VE-cadherin	BBB destructive	Li et al., 2020
	miR-101	VE-cadherin	BBB destructive	Mishra and Singh, 2013
	miR-132	EEF2K	BBB protective	Xu et al., 2017
	miR-210	Occludin/β-catenin	BBB destructive	Ma et al., 2017
Inflammation	miR-125a-5p	N/A	BBB protective	Reijerkerk et al., 2013
	miR-155	N/A	BBB destructive	Lopez-Ramirez et al., 2014
	miR-21-5p	TNF-α/IL-6	BBB protective	Ge et al., 2016
	miR-126-3p	VCAM-1	BBB protective	Fu et al., 2019
	miR-98 and let-7g*	CCL2 and CCL5	BBB protective	Rom et al., 2015
	miR-1303	MMP9	BBB protective	Lampugnani et al., 2018
	miR-132	MMP9	BBB protective	Li et al., 2018
Supporting cells coverage	miR-27b	SEMA6A/D	BBB protective	Demolli et al., 2017
	miR-150	Tie-2	BBB destructive	Fang et al., 2016
Apoptosis/cell cycle	miR-182	mTOR	BBB protective	Zhang et al., 2020
	miR-285	Yki/Mask	BBB protective	Li et al., 2017
Actin cytoskeleton	miRNA-181c	PDPK1	BBB destructive	Tominaga et al., 2015
Channel and receptor	miR-30a	ZnT4	BBB destructive	Wang et al., 2021
	miR-27a-3p	AQP11	BBB protective	Xi et al., 2018

During ischemic stroke, BBB integrity is compromised as shown by increased immune cell infiltration and solute leak, eventually leading to neuronal loss. In ischemic stroke, miR-15a/16-1 cluster was significantly upregulated to mediate BBB breakdown by direct downregulation of claudin-5. The specific depletion of the miR-15a/16-1 cluster in endothelial cells enhanced brain claudin-5 expression after transient ischemia in mice, resulting in the restoration of BBB integrity with smaller brain infarcts and decreased neuroinflammation (Ma et al., 2020). ZO-1 is another highly expressed tight junction molecule in the BBB Toyama et al. (2018). Identified miR-501-3p mediated inflammation-induced BBB breakdown via directly targeting ZO-1. Using a mouse model of vascular cognitive impairment with increased inflammation, they further showed that the expression of miR-501-3p and its target ZO1 were inversely correlated. Inhibition of miR-501-3p with a specific inhibitor rescued ZO-1 gene expression, leading to restoration of BBB integrity within the white matter and amelioration of working memory deficits.

The regulation of tight junctions by miRNA can also be indirect. For example, miRNAs can control the transcription factor of tight junction molecules to regulate their expression. One of the transcription factors of claudin-5 is forkhead box protein O1 (FoxO1), which positively regulates claudin-5 expression (Taddei et al., 2008). By directly targeting FoxO1, miR-182 negatively regulates claudin-5 expression and tight junctions of brain endothelial cells, while inhibition of miR-182 protects BBB integrity (Zhang et al., 2020). MiR-107 was identified to directly target endophilin-1 (Liu et al., 2016), which regulates ZO-1 and occludin expression via the epidermal growth factor receptor (EGFR)-extracellular signal-regulated protein kinase (ERK)1/2 pathway (Liu et al., 2014). In addition, miR-143 was shown to contribute to methamphetamine-induced BBB disruption by targeting p53 unregulated modulator of apoptosis (PUMA), which leads to a decrease of tight junction molecules, such as claudin-5, occludin, and ZO-1 (Bai et al., 2016).

Adherens junctions form before tight junctions and are also a major regulator in vascular integrity (Taddei et al., 2008; Lampugnani et al., 2018). VE-cadherin, a key adherens junction molecule, was shown to be decreased in cerebral vascular diseases (Li et al., 2020) and neurodegenerative diseases (Li et al., 2018). MiR-27a directly regulates VE-cadherin (Young et al., 2013). In cerebral cavernous malformation, where BBB is disrupted, blockage of the interaction between miR-27a and VE-cadherin by a specific target site blocker CD5-2 restored BBB integrity and reduced severity of diseases (Li et al., 2020). VE-cadherin was also shown to be regulated by miR-101 (Mishra and Singh, 2013). In HIV-1-infected human brain microvascular endothelial cells, miR-101 mediated the disruptive effect of infection on endothelial barrier integrity by downregulating VE-cadherin. MiRNA may also indirectly regulate VE-cadherin. For example, miR-132 directly targets eukaryotic elongation factor 2 kinase (EEF2K), which inhibits VE-cadherin by phosphorylation of eukaryotic elongation factor 2 (eEF2) (Xu et al., 2017). In this interesting study, neurons secreted miR-132-containing exosomes to brain endothelial cells, leading to an increase in functional and mature miR-132 expression in brain endothelial cells. Consequently, VE-cadherin was upregulated, and BBB integrity was enhanced. When miR-132 was antagonized by specific miR-132 morpholino antisense oligonucleotides, severe intracranial hemorrhage and disrupted BBB integrity was exhibited. Although VE-cadherin is an upstream regulator of claudin-5 and tight junctions (Taddei et al., 2008), in this study, changes in tight junction molecules such as claudin-5, occludin, ZO-1, were not observed, suggesting VE-cadherin can modulate BBB integrity via modulating adherens junctions without altering tight junctions.

Some miRNAs regulate BBB integrity by targeting both tight and adherens junction molecules. For example, miR-210 was shown to directly regulate tight junction molecule occludin and adherens junction molecule β-catenin (Ma et al., 2017). During ischemia, miR-210 expression was significantly upregulated in the brain. Inhibition of miR-210 with its complementary locked nucleic acid oligonucleotides (miR-210-LNA) reduced BBB leakiness by increasing expression of both occludin and β-catenin in the BBB.

### MiRNAs and Inflammation

Inflammation is often associated with disrupted BBB (Haruwaka et al., 2019). On the one hand, miRNAs can mediate inflammation-induced BBB permeability; on the other hand, miRNAs may act as an upstream of inflammation by targeting inflammation molecules and pathways to regulate BBB integrity (Table 1).

In brain endothelial cells treated with the proinflammatory mediator tumor necrosis factor α/interferon γ (TNFα/IFNγ), 107 miRNAs were significantly changed (Reijerkerk et al., 2013), among which miR-125a-5p was downregulated. Consistently, in the inflamed blood vessels of patients with multiple sclerosis (MS), there was also significantly less miR-125a-5p than in its expression in non-inflamed blood vessels (Reijerkerk et al., 2013), suggesting a positive correlation between miR-125a-5p and BBB integrity during inflammation. In contrast, miR-155 was shown to negatively affect BBB integrity during inflammation (Lopez-Ramirez et al., 2014). It was upregulated in disrupted BBB of MS human patients and animals—in experimental allergic encephalomyelitis (EAE), a model of MS. When miR-155 was knocked out in EAE mice, BBB leakage was reduced by 50% compared with wild-type mice. Inhibition of miR-155 also reduced TNFα/IFNγ-induced endothelial permeability *in vitro*. The putative targets of miR-155 included focal adhesion molecules and junctional complex, suggesting miR-155 may function by targeting these molecules to modulate BBB integrity.

MicroRNAs can also target inflammatory cytokines or markers to regulate BBB integrity, as exemplified by miR-21-5p, which regulates the BBB by targeting pro-inflammatory cytokines TNF-α, interleukin 6 (IL-6), and nuclear factor kappa B (NF-κB) signaling (Ge et al., 2016). Another miRNA, miR-126, was shown to attenuate intracerebral hemorrhage-induced leukocyte adhesion and BBB disruption by targeting vascular cell adhesion molecule-1 (VCAM-1), a classic inflammation marker critical for leukocyte adhesion to blood vessels (Fu et al., 2019). Glycogen synthase kinase 3β (GSK3β) was shown to protect BBB under neuro-inflammation conditions. MiR-98 and let-7g^∗^, both of which belong to the highly conserved let-7 family, mediated the BBB-protective effect of GSK3β by targeting inflammatory molecules CCL2 and CCL5 (Rom et al., 2015). Overexpression of let-7g^∗^ and miR-98 reduced neuro-inflammation-induced BBB leakiness. Matrix metalloproteinase-9 (MMP9) contributes to inflammation-induced BBB breakdown (Shigemori et al., 2006; Turner and Sharp, 2016). Several miRNAs, including miR-1303 and miR-132, have been shown to target MMP9 and play a protective role in BBB integrity under inflammation conditions (Song et al., 2018; Zuo et al., 2019).

### MiRNAs and Crosstalk Between Brain Endothelial Cells and Supporting Cells

Pericyte coverage on brain endothelial cells is another key indicator of BBB integrity (Ting et al., 2019). When pericyte coverage is reduced, BBB permeability is increased. MiRNAs can modulate the integrity of the BBB by regulating pericyte coverage-associated molecules. One of the examples is miR-27. There are two miR-27s, miR-27a and miR-27b, which differ from each other by one nucleotide outside the seed region (Young et al., 2013). While miR-27a targets VE-cadherin to compromise BBB integrity, miR-27b promotes the interaction of endothelial cells with pericytes by targeting semaphorin 6A/D (SEMA6A/D), leading to enhancement of endothelial barrier function (Demolli et al., 2017; Table 1). This opposite role of miRNAs with identical seed regions is not rare, as evidenced by the difference in miR-23a and miR-23b (Li et al., 2016), reflecting the complexity of miRNA regulation.

The recruitment of pericytes to the endothelium can also be mediated by angiopoietins/Tie-2 signaling. MiR-150 was shown to target Tie-2 (Fang et al., 2016), leading to inhibition of claudin-5 expression and endothelial cell survival. It will be interesting to see whether miR-150 also has an effect on pericyte coverage to the BBB. Astrocytes, another type of BBB supporting cells, can release factors to strengthen BBB function via regulating miRNAs, one of which is mir-125a-5p, in the brain endothelial cells. How these miRNAs mediate crosstalk between astrocytes and brain endothelial cells remains to be defined (Reijerkerk et al., 2013).

### MiRNA and Other BBB-Relevant Pathways

In addition to the aforementioned endothelial tight junctions, inflammation and supporting cell coverage, other pathways regulating endothelial function, such as cell survival, cytoskeleton, and ion channels also contribute to the regulation of BBB integrity (Table 1).

Endothelial cell survival is critical for its function. MiR-182 was shown to mediate BBB breakdown in cerebral ischemia, a disease associated with massive BBB damage. Further studies suggested that miR-182 directly targeted mTOR, which is anti-apoptotic, suggesting miR-182 may regulate BBB integrity by regulating apoptosis of brain endothelial cells after ischemia. Mir-285 is another mRNA shown to affect BBB integrity through regulating apoptosis (Li et al., 2017). By targeting Yorkie (Yki in *Drosophila*, or YAP and TAZ in mammals), miR-285 inhibits cell proliferation and induces apoptosis, leading to regulation of BBB integrity.

The actin cytoskeleton is associated with endothelial barrier functions (Nag, 1995), through intercellular connections with tight and adherens junctions between endothelial cells. MiR-181C was shown to target 3-phosphoinositide-dependent protein kinase-1 (PDPK1), which delocalized actin fiber to destruct the BBB integrity. Intriguingly, unlike BBB-protective neuron-secreted miR-132, miR181c-containing extracellular vesicles can be secreted from brain metastatic cancer cells to break the BBB, leading to brain metastasis (Tominaga et al., 2015).

Several miRNAs have been shown to regulate BBB integrity by targeting channels and transporters in brain endothelial cells. MiR-30a targets zinc transporter ZnT4, leading to reduced intracellular free zinc in endothelial cells and an increase in BBB permeability in both cellular and animal models of ischemic stroke (Wang et al., 2021). In contrast, in intracerebral hemorrhage, miR-27a-3p protects against BBB disruption by targeting endothelial aquaporin-11 (AQP11), a functional water channel that permeates both water and glycerol with a possible role in the pathophysiology of brain edema (Xi et al., 2018). This is a conflict with the reports showing miR-27a-3p is disruptive for endothelial barrier function (Young et al., 2013; Li et al., 2020), indicating that a specific miRNA may function differently via regulating different targets.

## Challenges and Perspective

The effect of miRNAs on BBB integrity makes them a promising target to transiently open the BBB for brain-targeted drug delivery and to restore BBB integrity for disease treatment. However, several key challenges remain to be overcome before translating BBB-targeted miRNA-based therapeutics into the clinic (Rupaimoole and Slack, 2017).

The first challenge is the identification of miRNA targets. Through the miRNA array, the change in miRNA expressions caused by disrupted BBB can be measured (Reijerkerk et al., 2013). However, how these significantly regulated miRNAs contribute to BBB disruption remains unclear. This requires the identification of the targets of these miRNAs. This process often includes experiments to measure mRNA or protein levels of possible targets after modulating endogenous miRNA expression. To further identify whether the regulation is direct or indirect, luciferase reporter assays have been commonly used (Li et al., 2016). Because of the time for cloning and generation of mutants, these methods are quite time-consuming and only feasible for the identification of a small number of targets. In addition, it is becoming apparent that miRNAs may shift their targets in different types of cells and biological environments, further complicating the strategies for target validation.

The second challenge is the specificity of miRNA-based therapeutics. Each miRNA has dozens if not hundreds of potential targets. The ability of miRNAs to regulate a wide range of mRNAs gives them a unique advantage to regulate complex biological processes. However, it also raises possible side effects when miRNA expression is modulated. To overcome these drawbacks, antisense oligonucleotides (ASOs) that specifically block miRNA interaction with a mRNA of interest have been developed (Young et al., 2013; Zhao et al., 2017). Instead of modulating miRNA expression, these ASOs, named miR-Mask, target site blocker (TSB), or BlockmiRs, bind to the miRNA binding sites in the 3′UTR of the target mRNA through full complementarity (Sonneville et al., 2017; Natarelli et al., 2018). Consequently, they prevent miRNA from regulating a specific mRNA. This can be highly useful in identifying the importance of a specific miRNA:mRNA interaction or developing miRNA therapeutics for validated drug targets. However, the design of TSB remains a challenge as the principles of the design are not fully understood. Further understanding and improvement in the design principles are required to improve the success rate.

The third challenge is the delivery of miRNA-based therapeutics to the brain. Current modifications of miRNA modulators, including mimics and inhibitors, have successfully increased their retention time in the circulation system (Rupaimoole and Slack, 2017). However, the majority of naked miRNA modulators are accumulated in the liver and kidneys. It is important to increase miRNA accumulation in the brain to enhance their effect targeting the BBB. Viral and non-viral delivery systems with specificity to brain endothelial cells have been successfully employed to deliver nucleotide-based drugs into the brain (Lee et al., 2019; Marcos-Contreras et al., 2020). However, the potential immunostimulatory effects and toxicity of these delivery systems may hinder clinical translation (Mitchell et al., 2021). Identification of high-affinity ligands targeting BBB-specific receptors and development of biocompatible delivery materials are required to improve the specificity and efficiency of BBB-targeted delivery systems.

## Author Contributions

JW and JL contributed to the conception and design of the review and wrote the first draft of the manuscript. FX, XZ, XL, and YL critically revised the manuscript. All authors contributed to the article and approved the submitted version.

## Conflict of Interest

The authors declare that the research was conducted in the absence of any commercial or financial relationships that could be construed as a potential conflict of interest.
